# Assessment of Extrapulmonary Tuberculosis Using Gene Xpert MTB/RIF Assay and Fluorescent Microscopy and Its Risk Factors at Dessie Referral Hospital, Northeast Ethiopia

**DOI:** 10.1155/2018/8207098

**Published:** 2018-08-07

**Authors:** Yeshi Metaferia, Abdurahaman Seid, Genet Mola Fenta, Daniel Gebretsadik

**Affiliations:** Department of Medical Laboratory Science, College of Medicine and Health Sciences, Wollo University, Ethiopia

## Abstract

**Background:**

Tuberculosis is a major public health problem and extrapulmonary tuberculosis (EPTB) accounts for a significant proportion of tuberculosis cases worldwide.

**Objective:**

To determine the magnitude of EPTB, associated risk factors, and agreement of diagnostic techniques at Dessie Referral Hospital.

**Methods:**

A cross-sectional study was conducted on consecutive presumptive EPTB cases from March 1 to June 30, 2017. Sociodemographic characteristics and other variables were collected using a structured questionnaire. Clinical specimens were collected and processed using fluorescent microscopy and Gene Xpert assay. Data was analyzed using SPSS version 20. Chi-square test and logistic regression were done and a P value of ≤0.05 was taken as statistically significant.

**Results:**

From a total of 353 presumptive EPTB cases the overall prevalence of Gene Xpert assay and smear confirmed patients was 8.8% and 2.5%, respectively. Tuberculosis lymphadenitis was the predominant (33.3%) type followed by pleural (11.9%) and peritoneal (6.7%) tuberculosis. Previous history of pulmonary tuberculosis was significantly associated with extrapulmonary infection (AOR:2.8; 95%CI: 1.05-7.54; p=0.04); however, other variables such as age, residence, sex, marital status, occupation, level of education, and monthly income did not show any association.

**Conclusion:**

High proportions (71%) of Gene Xpert assay confirmed EPTB patients were smear-negative. Sensitivity of microscopy should be enhanced in resource limited countries like Ethiopia where Gene Xpert machine is not easily accessible.

## 1. Introduction

Tuberculosis (TB) is a chronic infectious disease and is a major public health problem [1, 2]. It is the ninth leading cause of death worldwide and the leading cause from a single infectious agent. Globally, according to 2017 World Health Organization (WHO) report, there were an estimated 10.4 million TB cases and 1.6 million TB deaths in 2016 [1], which is much higher than 2013 estimate (9.0 million TB cases and 1.5 million TB deaths) [2]. The Majority of TB incident cases occurred in the Southeast Asia Region (45%) as well as the African (25%) and Western Pacific Region (17%). Among the 30 high TB burden countries Ethiopia stands 11th with an estimated TB incidence of 182 per 1000 individuals [1].

*Mycobacterium tuberculosis *(MTB) usually affects the lungs and causes pulmonary tuberculosis (PTB). Majority of estimated number of incident TB cases were PTB (85%) [1]. However, the bacterium can also affect extrapulmonary sites such as lymph nodes, pleura, abdomen (peritoneum and gastrointestinal tract), bones and joints, genitourinary tract, central nervous system, and other multiple organs of the body and resulted in the development of extrapulmonary tuberculosis (EPTB) [3].

According to different reports, the incidence of EPTB has been increasing among TB patients across Ethiopia since the 1990s and recently the estimated numbers of EPTB cases reached 32% in the country which is higher than other African countries [1]. This is mostly associated with high HIV epidemic in the country [4]. Furthermore, EPTB receives less attention than PTB because of its low infectious potential. The observed diverse manifestations of EPTB may also mimic other pathologies [5] and resulted in more diagnostic challenges, indirectly associated with diagnostic delay and giving it a greater potential for morbidity and mortality [6] when compared to PTB. Thus, EPTB deserves an increasing focus for proper management of TB cases and TB control program.

Besides regional variation of WHO report [1] the prevalence of EPTB varies across studies, and it depends on variation of the sociodemographic determinants, geographical location, study population, study designs, method of diagnosis, and rate of human immunodeficiency virus (HIV) coinfection [7]. Among patients with PTB the main determinants of EPTB were male sex, HIV infection, absence of fibrotic lung lesions, smear-negative PTB, anemia, and leukopenia [8]. Female sex, HIV infection, contact history with PTB cases, and low income level were also documented as independent risk factors of EPTB [9, 10].

A few cross-sectional studies were conducted among presumptive EPTB cases in Ethiopia for determination of risk factors and prevalence of EPTB which ranged from 9.9% to 29.8% [11, 12]. This is higher than other studies conducted elsewhere in the world (2-5%) [13–15]. Despite variation of EPTB diagnostic techniques, previous studies showed previous history of tuberculosis, contact with known tuberculosis cases, history of underlying chronic diseases, and income level as significant risk factors for EPTB [11, 12]. But, the agreement of the two most recently used TB diagnostic techniques (Gene Xpert MTB/RIF assay and fluorescent microscopy) was not assessed and evaluated. Besides EPTB risk factor analysis, evaluations of commonly used diagnostic tests are suggested among high TB burden countries like Ethiopia where resources are very limited [1]. Hence, this study was designed to determine the magnitude and risk factors of microbiologically confirmed EPTB and to compare diagnostic methods among presumptive EPTB cases in Dessie Referral Hospital, Northeast Ethiopia.

## 2. Materials and Methods

### 2.1. Study Design and Period

Hospital based cross-sectional study was conducted from March 1 to June 30, 2017.

### 2.2. Study Setting

The study was conducted at Dessie Referral Hospital which is located at South Wollo, Dessie town, Amhara Region, Northeast Ethiopia. Dessie is located in the Northeastern part of Ethiopia, 401 km from the capital city, Addis Ababa. The town's location is 11^0^8^1^N latitude and 39^0^38^1^E longitude. The hospital serves as a Referral Hospital with more than 300 beds and gives different inpatient and outpatient services to more than 7 million populations in the surrounding area and the adjacent regions.

### 2.3. Sample Size and Sampling Procedures

The study population comprised all clinically presumptive EPTB cases who visited adult outpatient departments (OPD and inpatient wards of Dessie Referral Hospital during the study period). The minimum representative sample size was determined using the single population proportion formula: N = z^2^ p (1-p)/ d^2^, where N is the number of presumptive EPTB cases; Z is standard normal distribution value at 95% CI which is 1.96; P is the prevalence of EPTB = 29.8% [12]; d is the margin of error taken as 5%. Accordingly, the estimated numbers of participants were 321. However, 353 inpatient and outpatient presumptive EPTB cases who visited the Referral Hospital during the study period were enrolled consecutively.

Presumptive EPTB case is any person who presents with signs and symptoms of EPTB including chronic lymphadenitis and body fluid accumulations like meningitis, pleural effusion, ascites, and others with clinical suspicion of tuberculosis. The definition of EPTB case is based on Ethiopian 2013 Ministry of Health guideline, and defined as TB in organs other than the lungs proven by a bacteriologically positive specimen from an extrapulmonary site. A bacteriologically confirmed case is one from whom a biological specimen is positive by smear microscopy, culture, or WHO-approved rapid diagnostics (such as Xpert MTB/RIF).

Presumptive EPTB cases aged ≥ 18 years and volunteering to participate were considered for inclusion, but presumptive cases who were critically ill and active pulmonary and extrapulmonary tuberculosis patients who were on anti-TB treatment were excluded from the study.

### 2.4. Data Collection and Quality Assurance

*Sociodemographic and Related Data.* Pretested structured questionnaire which was first prepared in English and then translated into the local language “Amharic version” was used to collect sociodemographic, lifestyle characteristics, and clinical history of the participants. Trained laboratory technologists working in TB laboratory division of Dessie Referral Hospital were employed as data collector.

*Clinical Sample Collection and Processing.* A total of three hundred and fifty-three extrapulmonary samples comprising cerebrospinal fluid (CSF) (184), peritoneal fluid (45), pleural fluid (109), and lymph node aspirates (15) were collected. All the samples were received in sterile containers. Fine needle aspirate (FNA) samples were collected by a pathologist, while other body fluid samples were collected by physicians during patient investigations and sent to the TB laboratory for microscopic investigation and Gene Xpert MTB/RIF assay analysis.

Before analysis, each sample was carefully homogenized and partitioned into two parts aseptically inside a biosafety cabinet (BSC). One of the split samples with small amount was used to prepare a smear on new, clean, unscratched frosted glass slide. Smears were fixed and stained with the staining reagents, auramine O stain (0.1% auramine O solution, 0.5% acid-alcohol, and 0.3% methylene blue). After drying, stained smears were examined under the light-emitting diode (LED) fluorescent microscopy (Primo Star iLED, Carl Zeiss, Gottingen, Germany) with 200x and 400x magnification for acid fast bacilli (AFB) [16].

For Gene Xpert MTB/RIF assay, one of the split samples with sufficient volume was treated with sample reagent (SR) containing NaOH and isopropanol as per the manufacturer's instruction [17]. Sample reagent was added in a 2:1 ratio to unprocessed sample in 15 ml falcon tube and the tube was manually agitated twice during a 15-minute incubation period at room temperature. From the treated sample 2 ml was transferred into multichambered plastic cartridge preloaded with liquid buffers and lyophilized reagent beads necessary for sample processing, deoxyribonucleic acid (DNA) extraction, and heminested real-time polymerase chain reaction (RT-PCR). The cartridge was loaded into the Xpert machine (GeneXpert®Dx System version 4.4a, Cepheid Company, 904 Caribbean Drive, CA 940889, USA), and an automatic process completes the remaining assay steps. The results were visualized and printable in the view results window.

*Quality Assurance.* The reliability of the study findings was guaranteed by implementing quality control measures throughout the whole processes of research activity. The questionnaire was pretested before the actual study began to make sure that the questions were appropriate and understandable. The collected data was checked daily for consistency and accuracy. BSC was used during sample preparation including initial homogenization, splitting, and smear preparation to ensure safety and avoid risk of contamination. The appropriateness of FM staining reagents was checked with a known positive and negative smear. Blind reading of the slide was performed by two independent laboratory technologists. Gene Xpert MTB/RIF assay has its own internal quality control system which was used during the investigation process. All laboratory tests were performed using standard operational procedures.

### 2.5. Data Management and Analysis

Data entry and analysis were made using SPSS version 20.0 statistical software. Descriptive statistics were used for analysis of patient characteristics. Chi-square tests and logistic regression analysis were done to determine the presence of a statistically significant association between explanatory variables and the outcome variable. To identify independently associated factors, multivariate logistic regression model was employed by taking presence of EPTB as an outcome variable. All explanatory variables that were associated with the outcome variable in the bivariate analysis (P ≤ 0.2) were included in the multivariate logistic regression model. Odds Ratio (OR), p value, and their 95% Confidence Intervals (CI) were calculated and the result was considered statistically significant at P ≤ 0.05.

### 2.6. Ethical Clearance

The Ethical Review Committee of Wollo University, College of Medicine and Health Sciences, approved the study. A permission and support letter were also obtained from the management committee of Dessie Referral Hospital. Patients signed informed consent form to participate in the study. Information obtained at any course of the study was kept confidential. Positive results were made available to clinicians for decision-making as early as available.

## 3. Results

### 3.1. Sociodemographic, Lifestyle, and Clinical Characteristics

From a total of 353 presumptive EPTB cases who enrolled during the study period, majority (52.4%) were males. The age of the participants ranged from 18 to 77 years with mean of 38.16 years (± SD= 13.45 years) and 45.6% of study participants were in the age ranging from 31 to 50 years. About half (51.6%) of study participants were rural dwellers and 60.3% had a monthly income of less than 1500 Ethiopian birr. The majority (74.2%) of the study subjects were married and 40.0% were illiterate. About 61.2% of study participants had greater than three family members living together in the same room (Table 1).

Almost half of (49%) study participants had chronic diseases of whom 28.32% had diabetic mellitus (DM) followed by chronic kidney disease (21.96%) and hypertension (19.65%). However, lower proportion of study participants had previous history of PTB infection (9.1%) and contact with PTB patients (4.5%) and have had a habit of cigarette smoking (7.6%), alcohol drinking (14.7%), and consumption of raw milk (14.7%) (Table 2).

### 3.2. Prevalence of Gene Xpert and LED-FM Confirmed Mycobacterium Infection

Among all presumptive EPTB cases, the most commonly suspected site of infection was meninges (52.1%), followed by pleural cavity (30.9%) as shown in Table 3. The Gene Xpert MTB/RIF assay result showed that 31 out of 353 (8.78%) presumptive EPTB cases were positive for Mycobacterium tuberculosis complex. Of these, 9(2.5%) were AFB positive using LED-FM staining technique. The Gene Xpert MTB/RIF assay result revealed statistically significant variation among different types of body fluids (p= 0.001), but LED-FM technique did not show any significant variation (p>0.05). Higher discordant results between LED-FM and Gene Xpert MTB/RIF assay were observed. For instance, out of the thirteen Gene Xpert MTB/RIF assay positive pleural fluid samples only two were positive for AFB by LED-FM technique as shown in Table 3. Cohen's Kappa was computed to evaluate the agreement between Gene Xpert MTB/RIF assay and LED-FM microscopy. The result showed that the test agreement of the two methods was 0.427 indicating that these two methods do not provide similar results on the diagnosis of EPTB infection.

### 3.3. Associated Risk Factors of EPTB Infection

In the current study, sociodemographic characteristics such as age, sex, residence, monthly income, family size living together, occupation, and educational level were not significantly associated with EPTB. Furthermore, bivariate analysis also showed that lifestyle characteristics such as cigarette smoking, alcohol drinking, and consumption of raw milk were not significantly associated with EPTB. However, history of previous PTB infection (COR: 2.73; 95%CI: 1.03-7.26) and contact with PTB patients (COR: 3.83; 95%CI: 1.16-12.68) were significantly associated with EPTB Gene Xpert MTB/RIF assay positivity. On multivariate logistic regression analysis, previous history of PTB (AOR: 2.81; 95%CI: 1.05-7.54) remains the only predictor of EPTB (Table 4).

## 4. Discussion

Nowadays EPTB is becoming a major concern of tuberculosis control programs. Formerly, it was more prevalent in developed nations than developing countries but these days it has high proportion in developing nations including Ethiopia [1]. Identification of the risk factors that predispose to EPTB would allow designing targeted strategies to prevent active tuberculosis infection and hence decrease the prevalence of EPTB. The current study was conducted among presumptive EPTB cases for determination of EPTB prevalence and risk factors associated with it.

In this study, the most common suspected extrapulmonary sites were meninges, pleural, peritoneal, and lymph nodes in decreasing order. However, TB disease detection rate was highest among patients suspected of having TB lymphadenitis (33.3%) followed by pleural effusion (11.9%) as shown in Table 3. Our result is in line with previous study as TB lymphadenitis is diagnosed among 33.3 to 82.4% of presumptive EPTB cases in Ethiopia [11, 12]. Similarly, studies conducted in other countries showed TB lymphadenitis as the common form of EPTB followed by pleural forms [18–20]. Recent reports from Sudan also indicated that TB lymphadenitis is the most frequent form of EPTB followed by peritoneal TB [21]. In contrast, other reports have shown pleural effusion as the most frequent form of EPTB, followed by lymphatic TB [22, 23]. The reasons for these discrepancies were unknown; however, authors considered pleural TB as an early manifestation of primary mycobacterial infection [24].

In this study the overall prevalence of Gene Xpert MTB/RIF assay positive EPTB cases was 8.8% which is lower than study conducted in Gondar, Ethiopia (26.2%) [12]. The low prevalence rate of EPTB in our study cohort might be due to earlier presentation of presumptive cases with more paucibacillary disease, which might result in a decreased sensitivity of Gene Xpert MTB/RIF assay [25]. Furthermore, in our study, the prevalence of smear-positive (LED-FM) EPTB cases was 2.5% which is lower than the prevalence rate reported in Ethiopia (9.9%) [11], Nigeria (5%) [14], and India (3.9%) [15]. But our smear detection rate is comparable with reports of Patel et al., 2.26% [13]. The observed difference might be due to variation of healthcare providers' knowledge of EPTB suspicion and diagnosis.

Moreover, it could be attributed to difference in sociodemographic characteristics of study participants and variation in study design or laboratory diagnostic techniques. For instance, studies conducted in Gondar, Ethiopia, utilized culture and concentrated specimens for Gene Xpert MTB/RIF assay [12] and smear microscopy [11], whereas in our study due to lack of infrastructure direct samples were used for Gene Xpert MTB/RIF assay and smear microscopy. In line with this evidence, Tadesse and colleagues demonstrated that bleach concentration and pelleting of smear-negative samples increase Gene Xpert MTB/RIF sensitivity from 63.2% to 73.8% [26]. This, in fact, highlights the benefit of concentrating nonrespiratory samples for diagnosis of EPTB using Gene Xpert MTB/RIF assay and microscopy; however, it needs comprehensive and comparative study, particularly in resource limited countries like Ethiopia, to elucidate the long term benefit of nonrespiratory sample concentration for TB patient management.

In our study, Gene Xpert MTB/RIF assay analysis also showed significant variation among nonrespiratory samples to detect Mycobacterium tuberculosis complex (p=0.001) but no statistical difference was observed using LED-FM (Table 3). The Mycobacterium tuberculosis complex detection rate was 33.3% for lymph node aspirates and 11.9% for pleural fluid. It is already documented that Gene Xpert MTB/RIF assay sensitivity differed substantially between sample types. A recent systematic review and meta-analysis showed that Gene Xpert MTB/RIF assay was highly sensitive for TB detection in lymph node samples and moderately sensitive for the detection of TB meningitis (83.1% and 80.5%, respectively), but lower sensitivity was shown (46.4%) for testing pleural fluid [27].

In our analysis, Gene Xpert MTB/RIF assay and LED-FM did not provide similar results with 0.427 Cohen's Kappa measure of agreement. However, all samples which were LED-FM smear-positive were also Gene Xpert MTB/RIF assay positive. This is due to the high sensitivity (97.4%) of Gene Xpert MTB/RIF assay in smear-positive samples across all sample types [25]. Additionally, Bates et al. in their study reported a better detection rate with Gene Xpert MTB/RIF assay when compared to smear microscopy and culture [28], and Xpert MTB/RIF analysis is found to increase the TB detection rate by 47.4% compared with smear microscopy [29]. Lombardi et al. also showed a 73.1% Gene Xpert MTB/RIF assay sensitivity in smear-negative samples as compared to culture which was independent of specimen origin [30]. A systematic review showed a 66% performance sensitivity of Gene Xpert MTB/RIF assay for diagnosis of smear-negative nonrespiratory samples [31].

In the current study, the highest proportion (about 71%) of smear-negative but Gene Xpert MTB/RIF assay positive results could be partially ascribed to performing smear microscopy directly on unprocessed sample. In our cohort, although the performance of smear microscopy was clearly inferior to that of Xpert MTB/RIF, no false positive smear results were observed. In our opinion, Xpert MTB/RIF assay should be considered as an adjunct test for improved case detection in smear-negative EPTB suspects in low income settings like Ethiopia, instead of replacing smear microscopy.

In the current study, sociodemographic characteristics like sex, age, marital status, and occupation were not significantly associated with EPTB infection; similar findings were reported in Gonder, Ethiopia [11]. Additionally, univariate analysis showed that history of contact with pulmonary TB cases was a significant risk factor for EPTB but if adjusted for other variables it lost its association. The most important risk factor that was significantly associated with EPTB infection in the present study was history of previous PTB infection (P=0.04) which is similar to previous reports [9, 10]. A study conducted in Cameron showed that about a quarter of patients with PTB have developed EPTB and both HIV infection and smear-negative PTB emerged as independent predictors of extrapulmonary involvement [8].

Our results also demonstrated that smoking was not associated with EPTB which is consistent with other studies [21] and smoking was reported as the risk factor for PTB compared with EPTB [32]. Similarly, in our study occupation and residence of participants were not associated with EPTB which is in agreement with study conducted in Sudan [21]. In the present study, only two samples demonstrated rifampicin resistant Mycobacterium tuberculosis complex making the prevalence of rifampicin resistant-TB 6.5% (2/31). Report from Addis Ababa showed a 2.3% MDR-TB among newly diagnosed EPTB cases [33]. In the Northwestern Ethiopia, the prevalence of MDR-TB among EPTB cases was found to be 3.7% [34]. Even though the observed frequency of rifampicin resistant case is low, it highlights the need of screening of EPTB patient for MDR-TB.

Our study has some limitations. Firstly, being conducted in hospital might not reflect reality of the community but it gives valuable information on burden of EPTB as well as importance of MDR-TB screening of EPTB cases in the study area. Although HIV infection is known risk factor for increasing EPTB infection, HIV status was not known in substantial number of EPTB suspected cases. However, this is quite important to be investigated with another future research. The present study employed only fluorescence staining technique and Gene Xpert MTB/RIF assay which have generally low sensitivity in comparison with culture, which is the gold standard for detection of EPTB due to paucibacillary nature of specimens and thus may underestimate the prevalence EPTB among the study cohorts.

## 5. Conclusions

The prevalence of Gene Xpert MTB/RIF assay and LED-FM confirmed EPTB was 8.8% and 2.5%, respectively. The most prevalent type of EPTB was TB lymphadenitis. Sociodemographic characteristics and lifestyle factors did not show significant association with EPTB. However, a previous history of pulmonary tuberculosis infections was significantly associated with Gene Xpert MTB/RIF assay confirmed EPTB. Discordant results were observed between LED-FM and Gene Xpert MTB/RIF assay with 71% (22/31) of smear-negative EPTB cases being Gene Xpert MTB/RIF assay positive and this needs developing strategies to enhance the sensitivity of FM microscopy for diagnosis of EPTB in resource limited countries like Ethiopia. Moreover, large scale study on the prevalence and risk factor of EPTB infection using culture or other more sensitive methods could help to determine the exact prevalence of EPTB infection in the study area.

## Figures and Tables

**Table 1:** Sociodemographic characteristics of presumptive EPTB cases at Dessie Referral Hospital, from March 1 to June 30, 2017.

**Variable **	Frequency	Percent
** Age in years**		
18-30	132	37.4
31-50	161	45.6
>50	60	17.0
**Sex**		
Male	185	52.4
Female	168	47.6
**Residence**		
Urban	171	48.4
Rural	182	51.6
**Marital status**		
Married	262	74.2
Single	91	25.8
**Occupation **		
House wife	99	28.0
Merchant	84	23.8
Farmer	93	26.3
Others	77	21.8
**Educational Status**		
Illiterate	143	40.5
Grades 1-8	93	26.3
Grade 9 & above	117	33.1
**Monthly income**		
≤1500 birr	213	60.3
>1500 birr	140	39.7
**Family size**		
1-3	137	38.8
>3	216	61.2

**Table 2:** Lifestyle and clinical characteristics of presumptive EPTB cases at Dessie Referral Hospital, from March 1 to June 30, 2017.

Variable	Frequency	Percent
**Cigarette Smoking **		
Yes	27	7.6
No	326	92.4
**Alcohol Drinking **		
Yes	52	14.7
No	301	85.3
**previous PTB infection**		
Yes	32	9.1
No	321	90.9
**chronic illness**		
Yes	173	49.0
No	180	51.0
** Type chronic illness (n=173)**		
Kidney	38	21.96
HIV	32	18.49
DM	49	28.32
Hypertension	34	19.65
Others	20	11.56
**Contact with PTB patients**		
Yes	16	4.5
No	337	95.5
**Consumption of raw milk**		
Yes	52	14.7
No	301	85.3

**Table 3:** Prevalence of Gene Xpert MTB/RIF assay and LED-FM confirmed EPTB patients at Dessie Referral Hospital, from March 1 to June 30, 2017.

**Sample type **	**Total **	**Gene Xpert MTB/RIF assay**		*χ*2	**LED- FM**		*χ*2
	**n(**%**)**	**Negative** n(%)	**Positive** n(%)	p-value	**positive** n(%)	**negative** n(%)	p-value
CSF	184(52.1)	174 (94.6)	10 (5.4)	**15.457;** **0.001**	6(3.3)	178 (96.7)	**2.799;** **0.424**
Peritoneal fluid	45(12.7)	42(93.3)	3(6.7)		0(0)	45(100)	
Pleural fluid	109(30.9)	96 (88.1)	13(11.9)		2(1.8)	107 (98.2)	
LNA	15(4.3)	10 (66.7)	5 (33.3)		1(6.7)	14 (93.3)	
**Total **		322(91.22)	31(8.78)		9(2.5)	344(97.5)	

CSF: cerebrospinal fluid; LNA: lymph node aspirate.

**Table 4:** Factors associated with Gene Xpert MTB/RIF assay positivity among presumptive EPTB cases at Dessie Referral Hospital, from March 1 to June 30, 2017.

**Variable **	Freq	Xpert result		COR (95%CI)	P-value	AOR (95%CI)	p-value
		Negative=n (%)	Positive=n (%)				
**Age in years**							
18-30	132	119(90.2)	13(9.8)	ref			
31-50	161	147(91.3)	14(8.7)	0.87(0.40-1.93)	0.734		
>50	60	56(93.3)	4 (6.7)	0.65(0.20-2.10)	0.475		

**Sex**							
Male	185	170(91.9)	15(8.1)	ref			
Female	168	152(90.5)	16(9.5)	1.19(0.57-2.49)	0.639		

**Residence**							
Urban	171	161(94.2)	10(5.8)	ref			
Rural	182	161(88.5)	21(11.5)	2.1(0.95-4.60)	0.064	2.14(0.97-4.71)	0.059

**Marital status**							
Married	262	238(90.8)	24(9.2)	ref			
Single	91	84(92.3)	7(7.7)	0.83(0.34-1.99)	0.670		

**Occupation **							
House wife	99	88(88.9)	11(11.1)	ref			
Merchant	84	79(94.0)	5(6.0)	0.51(0.17-1.52)	0.225		
Farmer	93	83(89.2)	10(10.8)	0.96(0.39-2.39)	0.937		
Others	77	72(93.5)	5(6.5)	0.56(0.19-1.67)	0.296		

**Educational Status**							
Illiterate	143	128(89.5)	15(10.5)	1.84(0.73-4.68)	0.199		
Grades 1-8	93	84(90.3)	9(9.7)	1.68(0.60-4.71)	0.320		
Grade 9 & above	117	110(94.0)	7(6.0)	ref			

**Monthly income**							
<1500 birr	213	191(89.7)	22(10.3)	1.68(0.75-3.76)	0.209		
>1500 birr	140	131(93.6)	9(6.4)	ref			

**Family size**							
<4	137	125(91.2)	12(8.8)	ref			
4 & above	216	197(91.2)	19(8.8)	1.0(0.47-2.14)	0.990		

**Cigarette Smoking**							
Yes	27	22(81.5)	5(18.5)	2.6(0.92-7.50)	0.072		
No	326	300(92.0)	26(8.0)	ref			

**Alcohol drinking**							
Yes	52	45(86.5)	7(13.5)	1.8(0.73-4.41)	0.20		
No	301	277(92.0)	24(8.0	ref			

**Previous PTB**							
Yes	32	26(81.2)	6(18.8)	2.73(1.03-7.26)	0.044	2.8(1.05-7.54)	0.040
No	321	296(92.2)	25(7.8)	ref			

**Chronic disease**							
Yes	173	156(90.2)	17(9.8)	1.29(0.62-2.71)	0.497		
No	180	166(92.2)	14(7.8)	ref			

**Contact with PTB patients**							
Yes	16	12(75.0)	4(25.0)	3.83(1.16-12.68)	0.028		
No	337	310(92.0)	27(8.0	ref			

**Consumption of raw milk**							
Yes	52	48(92.3)	4(7.7)	0.86(0.28-2.53)	0.764		
No	301	274(91.0)	27(9.0)	ref			

COR: crude odds ratio; AOD: adjusted odds ratio.

## Data Availability

The data used to support the findings of this study are included within the article.

## Abbreviations

MTB: Mycobacterium tuberculosis
TB: Tuberculosis
EPTB: Extrapulmonary tuberculosis
PTB: Pulmonary tuberculosis
LED: Light-emitted diode
FM: Fluorescent microscope.
